# Development of birth weight estimation model for Ethiopian population from sonographic evaluation

**DOI:** 10.1186/s12884-023-06145-9

**Published:** 2023-12-11

**Authors:** Nejat Mohammed Seman, Hamdia Murad Adem, Fanta Assefa Disasa, Gizeaddis Lamesgin Simegn

**Affiliations:** 1 Biomedical Imaging Unit, School of Biomedical Engineering, Jimma Institute of Technology Jimma University, Jimma, Ethiopia; 2 Department of Obstetrics and Gynecology, Jimma Institute of Health Sciences, Jimma University, Jimma, Ethiopia

**Keywords:** FBW estimation, Sonographic evaluation, Mathematical model, Multiple linear regression

## Abstract

**Background:**

Fetal birth weight (FBW) estimation involves predicting the weight of a fetus prior to delivery. This prediction serves as a crucial input for ensuring effective, accurate, and appropriate obstetric planning, management, and decision-making. Typically, there are two methods used to estimate FBW: the clinical method (which involves measuring fundal height and performing abdominal palpation) or sonographic evaluation. The accuracy of clinical method estimation relies heavily on the experience of the clinician. Sonographic evaluation involves utilizing various mathematical models to estimate FBW, primarily relying on fetal biometry. However, these models often demonstrate estimation errors that exceed acceptable levels, which can result in inadequate labor and delivery management planning. One source of this estimation error is sociodemographic variations between population groups in different countries. Additionally, inter- and intra-observer variability during fetal biometry measurement also contributes to errors in FBW estimation.

**Methods:**

In this research, a novel mathematical model was proposed through multiple regression analysis to predict FBW with an accepted level of estimation error. To develop the model, population data consisting of fetal biometry, fetal ultrasound images, obstetric variables, and maternal sociodemographic factors (age, marital status, ethnicity, educational status, occupational status, income, etc.) of the mother were collected. Two approaches were used to develop the mathematical model. The first method was based on fetal biometry data measured by a physician and the second used fetal biometry data measured using an image processing algorithm. The image processing algorithm comprises preprocessing, segmentation, feature extraction, and fetal biometry measurement.

**Results:**

The model developed using the two approaches were tested to assess their performance in estimating FBW, and they achieved mean percentage errors of 7.53% and 5.89%, respectively. Based on these results, the second model was chosen as the final model.

**Conclusion:**

The findings indicate that the developed model can estimate FBW with an acceptable level of error for the Ethiopian population. Furthermore, this model outperforms existing models for FBW estimation. The proposed approach has the potential to reduce infant and maternal mortality rates by providing accurate fetal birth weight estimates for informed obstetric planning.

## Introduction

FBW is an important indicator for the optimal growth, survival, and future well-being of newborns. A normal size infant is one weighing greater than 2500 g and less than 4000 g. Low birth weight is between 1500 and 2500 g. A birth weight between 1000 and 1500 g is considered a very low birth weight. Below 1000 g is an extremely low birth weight and more than 4000 g is a high birth weight or macrosomia. So, FBW estimation is to estimate these values before birth when the infant is inside his/her mother’s womb. Maternal ethnicity, infant sex, plurality, nutrition, altitude, education, and smoking affects the entire birthweight distribution in a country [1–7].

Globally 2.4 million children died in the first month of life in 2020 [8]. There are approximately 6700 newborn deaths every day, amounting to 47% of all child deaths under the age of 5 years [9]. In Africa, 1.12 million newborn deaths occur annually [10]. Preterm birth, intrapartum-related complications, infections, and birth defects cause most neonatal death, and our country Ethiopia is among the top 10 countries having the highest number (97 per 1000 live births) of newborn deaths, 2020 [10].

Birth weight estimation is an input for labor and delivery management plan which is used to determine the procedure taken during this period, so it is so important to know the birth weight of fetal before his/her birth date to overcome intrapartum-related complications associated with both giving birth to an infant having large weight [11] and small weight [12] which are greater than or equal to 4500 g and less than 2500 g respectively. Both extremely large and small fetal birth weights may lead to complications that cause lifetime impairment of body parts or death of the infant and mother. Regular and reliable birth weight estimation throughout the pregnancy period is vital to avoid those complications as early as possible.

There are two common methods to estimate FBW; clinical method and sonographic method. In the clinical method physicians measure the fundal height of the pregnant women then calculate FBW by using formula which is used to estimate FBW or perform abdominal palpation procedure to determine the fetal birth weight. In ultrasound machine there is a built-in software which calculate FBW. Estimation of FBW using ultrasound requires predefined formulae (model) which describes birth weight as dependent variable and some other variables like fetal biometry parameters as an independent variable. Several formulae [13–26] have been developed for estimating fetal weight by ultrasound. The most popular formulae are Shepard [21], Campbell [18], and Hadlock’s [17]. These formulae are included in most ultrasound equipment software packages. These formulae involve different types of fetal biometric parameters obtained by sonographic measurements. The measurement is taken by physicians during ultrasound examination. The techniques outlined for assessing FBW typically yield a reasonable margin of error. However, inaccuracies may arise due to factors such as insufficient expertise, subjectivity in assessing fetal biometry, fundal height, and abdominal palpation. It is worth noting that the mathematical models employed for birth weight estimation are derived from populations in other countries, thereby resulting in an estimation error of over 10% for Ethiopian births when utilizing such models [27]. A birth weight estimate with an error margin of 10% or less is deemed acceptable [28, 29].

Various mathematical models have been suggested for the estimation of FBW, as documented in scholarly research [28–32]. For example, a model was developed in Pakistan by S. Munim et al. [30] using the Regressions with Leaps and Bounds method based on population data. This model reported systematic and random errors of 10 and 250 g, respectively. Another study was conducted in India by S. Hiwale et al. [32] where multiple stepwise regression (MSR) and lasso regression methods were utilized to create population-based models with adjusted R^2^ values of 0.656 and 0.633, respectively. The accuracy of both models was determined to be 81% and 82% for estimating within ± 10% of the actual birth weight (ABW). Furthermore, C. Li et al. [33] proposed a gestational age stage-based birth weight prediction model for the Chinese population. The model employed multiple linear regression (MLR), fractional polynomial regression (FPR), and volume-based models (VM) to achieve systematic errors of 6.97%, 0.26%, and 0.36%, respectively.

A linear regression model was developed using obstetric factors (such as gravidity, gestational age, SFH, body mass index of the mother, membrane status, sex of the neonate, and actual birth weight) to estimate fetal weight by A. Yiheyis et al. [34]. Johnson’s formula was also evaluated to determine its suitability as a model for south western Ethiopia. R. Ramya et al. [31] utilized image processing algorithms on fetal ultrasound images to automatically measure fetal biometry, thereby increasing the accuracy of FBW estimation. The study involved measuring four major fetal biometrics (AC, HC, BPD, and FL) through different image processing steps. However, these techniques are found to be less accurate in estimation of fetal birth weights.

Deep learning techniques has been also employed in literatures for automatic estimation of fetal biometry [35–38]using ultrasound image or video data. These and the above techniques are not effective in estimating fetal births of the Ethiopian population. In order to address these issues, it is necessary to conduct population-specific measurements of parameters related to FBW. This will enable the development of effective and accurate models for estimating FBW, which will facilitate proper planning and management of labor and delivery. In this paper we propose the use of an automatic image processing algorithm for measuring fetal biometry to develop a mathematical model that accurately estimates FBW based on our Ethiopian population.

## Materials and methods

**Fig. 1:**
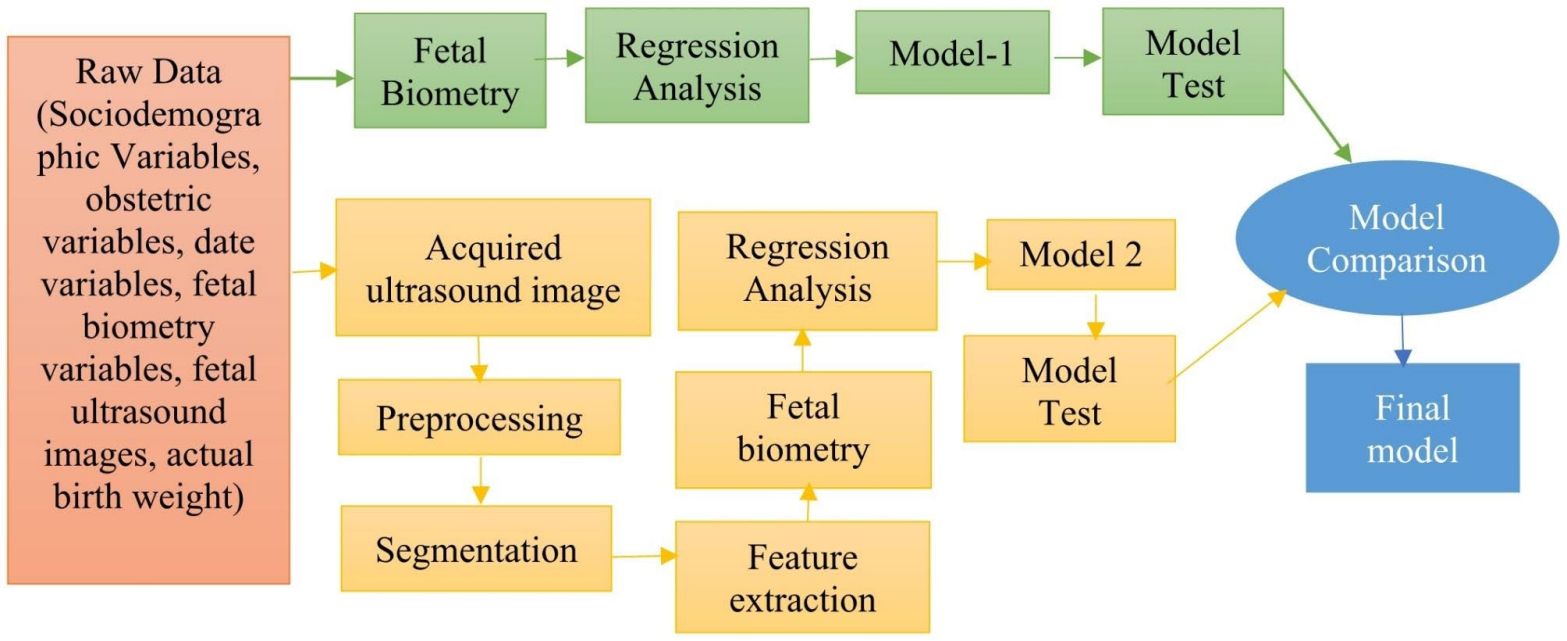
Block diagram of the model development

The proposed FBW estimation model was developed by using multiple linear regression analysis through two different approaches. The main difference between the approaches lies in the measurement of fetal biometrics. Figure 1 illustrates the procedures of model development using two approaches.

### Data collection

#### Study population, sampling, inclusion and exclusion criteria

The study population include pregnant women who underwent ultrasound examinations between June 2021 and August 2021 at Shenen Gibe Hospital and FGAE (Family Guidance Association of Ethiopia), as well as between September 2021 and January 2022 at Zeweditu Memorial Hospital and Abebech Gobena MCH Hospital. The study utilized a cross-sectional research design to gather data, and the study sample comprised pregnant women who satisfied the following inclusion criteria:

##### Inclusion criteria

30–42 gestational week pregnant women who underwent ultrasound evaluation during study time in study conducted health facilities.

##### Exclusion criteria

Abortus, known severe fetal congenital anomalies, polyhydramnios (amniotic fluid index greater than 24 cm or clinically assessed), known fibroid or congenitally abnormal uterus.

##### Study sample size

The sample size of the study was determined using the following single population estimation formula (Eq. (1)) [34].

1 $$N = P(1 - P){Z^2}/{d^2}$$

The following assumptions were used in determining the sample size:

- P – prevalence of subject in the population (in our case it is unknown so we take P = 0.5).
- Z = 1.96 which is the standard normal variable at 95% confidence level.
- d-is the margin of sampling error tolerated = 5%.
  - So, N = 0.5(1-0.5)1.96^2^/ (0.05)^2^ =384.
  - 384 mothers are needed to give a precision of 5% around an observed percentage of estimated fetal weights correct to within 10% estimation error of the birth weight.

#### Study variables

During the ultrasound evaluation of pregnant women, fetal biometry variables and image data were obtained using an ultrasound machine to develop the model. Additionally, data was collected on obstetric factors such as gestational age, fetal sex, and actual birth weight, as well as sociodemographic factors such as ethnicity, age, marital status, educational background, income, and area of residence. Date variables including expected delivery date, actual delivery date, and ultrasound examination date were also recorded. All variables, except for actual FBW, were considered independent variables in this study. Actual FBW was used as the dependent variable. Sociodemographic data was collected through the administration of a questionnaire after obtaining signed consent from the participants.

During the data collection process, a total of 484 pregnant women who underwent ultrasound examinations during the study period were enrolled from the following healthcare facilities: Shenengibe Hospital (20.8%), FGAE (Family Guidance Association of Ethiopia) Model Clinic (28.9%), Zeweditu Memorial Hospital (31.5%), and Abebech Gobena MCH (Maternal and Child Health) Hospital (18.8%). Out of 484 pregnant women, 384’s data were used to develop the model and the others are used for testing the model. In addition, 1,452 ultrasound images were collected. The ultrasound was performed within seven days of the delivery. The majority of women were between the age groups of 26–35 years with 52.6% percent from the total participant; 90.9% were married, collectively 77.4% of them were Oromo, Amhara and SNNPR in ethnicity, 61.7% of them finished their primary and secondary school, 66.9% were house wife. From the total participant 47.9% were from Jimma and 52.1% from Addis Ababa. The mean gestational age was 35.26 ± 3.04 weeks, with a range of 30–42 weeks. From the delivered infants 49.5% were females 63.2% have normal birth weight while 20.2 have low birth weight and 16.6% were macrocosmic. The mean birth weight was 3380.21 ± 418.84 g, with minimum of 2000 g and maximum of 4500 g.

### Data analysis

Pearson correlation analysis was used to investigate the strength of the linear relationship between the independent and dependent variables and their nature of association. Then regression models were used to describe those relationships between variables by fitting a line to the observed data. Regression allows you to estimate how a dependent variable change as the independent variable(s) change.

In multiple regression [39], the dependent or response variable y was predicted on the basis of an assumed linear relationship with several independent or predictor variables x_0_, x_1_, …, x_k_. In our study actual birth weight of fetal was the dependent variable and selected variables were an independent variable. The selection of the independent variable was done by correlation analysis. The multiple linear regression model can be expressed as in Eq. (2)

2 $$Y = \beta o + {\text{ }}{\beta _1}{x_1} + {\text{ }}{\beta _2}{x_2} + \ldots + {\text{ }}{\beta _k}{x_k} + \varepsilon$$

where: Y = response or prediction (in our case estimated FBW).

- βo, β1, β2…, βk are regression coefficients that are found after the statistical analysis.
- x_1_, x_2_…, x_k_ are predictors (in our case selected independent variables).
- ε = error (difference between actual FBW and estimated FBW).

## Results

### Model development based on fetal biometry measured by physician

After doing correlation analysis the selected variables as predictors or independent variables were maternal ethnicity, Fetal abdominal circumference and gestational age based on their Pearson correlation analysis coefficient. The multiple linear regression analysis was done to model the relationship between the dependent (actual birth weight) variable and independent variables (abdominal circumference, gestational age and maternal ethnicity). The model statistically significantly predicted birth weight with F (3, 380) = 22.001, p (0.000) < 0.05, R^2^ = 0.141 as shown in Tables 1 and 2. Also, the model level of prediction or R is equal to 38% with mean estimation of 3380 ± 161. 124.The final regression result was FBW can be estimated by using a linear Eq. (3). The model was tested on 85 cases that undergo ultrasound assessment before less than or equal to seven days before delivery. The absolute percentage error of the model was 7.53% and 70.61% estimation were with less than 10% percentage error (Tables 3 and 4).

3 $$EFW = 2294.857 + 81.018*AC - 42.132*GA + 13.970*E$$

**Table 1:** Model summary (predictors: constant, ethnicity, abdominal circumference, gestational age. dependent variable: actual birth weight)

Model summary					
Model	R	R square	Adjusted R square	Std. error of the estimation	Durbin-Watson
1.	0.385	0.148	0.141	388.135	1.689

**Table 2:** ANOVA table (dependent variable: actual birth weight. Predictors: (constant), ethnicity, abdominal circumference, gestational age). df: the degrees of freedom in the source, F-f-statistic

ANOVA						
Model		Sum of squares	DF	Mean square	F	Sig.
1.	Regression	9943087.524	3	3314362.508	22.001	0.000
	Residual	57246495.81	380	150648.673		
	Total	67189583.33	383			

**Table 3:** Coefficient table (dependent variable: actual birth weight, C = Constant, AC = abdominal circumference, GA = gestational age, E = ethnicity, T = tolerance, VIF = variance of inflation factor)

Coefficient								
Model		Unstandardized coefficients		Standardized coefficients	t	Sig.	Collinearity Statistics	
		B	Std. Error	Beta			T	VIF
1.	C	2294.857	231.543		9.911	0.000		
	AC	81.018	12.150	0.587	6.668	0.000	0.289	3.457
	GA	-42.132	12.156	− 0.306	-3.466	0.001	0.288	3.472
	E	13.970	6.540	0.102	2.136	0.033	0.991	1.009

**Table 4:** Mean percentage error of Model-1

Estimation	Range of percentage error	Count	Percent
Over estimation	10-15%	8	9.4%
	> 15%	4	4.7%
Estimation with accepted percentage error	< 10%	60	70.61%
Under estimation	10-15%	9	10.59%
	> 15%	4	4.7%
Total		85	100%

### Model development based on fetal biometry measured by image processing algorithm

#### Image processing for fetal biometry measurement

Automated image processing algorithm was developed to measurements four fetal biometry parameters: head circumference (HC), the biparietal diameter (BPD), the abdominal circumference (AC) and the femoral length (FL). This algorithm includes preprocessing, segmentation, feature extraction and fetal biometry measurement.

##### Image preprocessing

In the preprocessing stage acquired ultrasound image were changed to gray scale image (except for DICOM images) and denoised by using wavelet with soft thresholding denoising method. In the denoising process average peak signal to noise ratio (PSNR) of 59.42 dB and structural similarity index matrix (SSIM) of 0.9993 was achieved.

###### HC and BPD measurement

**Fig. 2:**
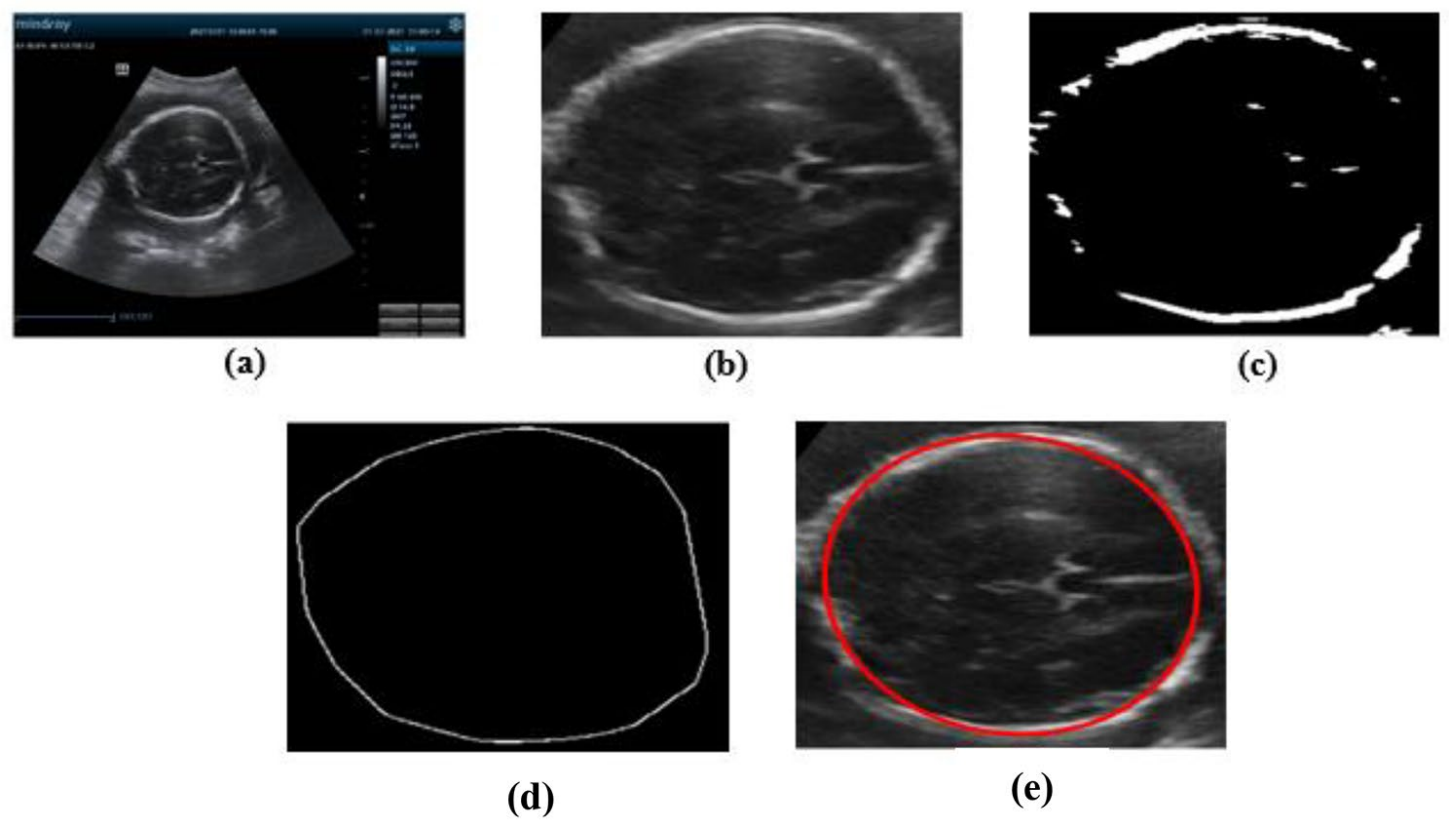
HC and BPD measurement steps, **(a)** original image, **(b)** denoised and cropped image, **(c)** segmented image, **(d)** convex hull then edge detected image, **(e)** ellipse fitted image

Head circumference and biparietal diameter of the fetal was measured from acquired ultrasound image using developed image processing algorithm. The first step was to change the image to gray scale image and denoising. Next segmented by adaptive thresholding technic to segment the image into background and foreground (edge of the head). Then convex hull morphological analysis and canny edge detector was used to connect the discontinues edge to form complete object. Finally, ellipse was fitted onto the object edge to measure HC and BPD. Analogously the circumference of the ellipse was HC and the minor axis of the ellipse was BPD. Outer-to-inner method was used to measure BPD. Figure 2 shows the result of the image processing algorithm to measure head circumference and biparietal diameter of the fetal from sample image.

###### AC measurement

**Fig. 3:**
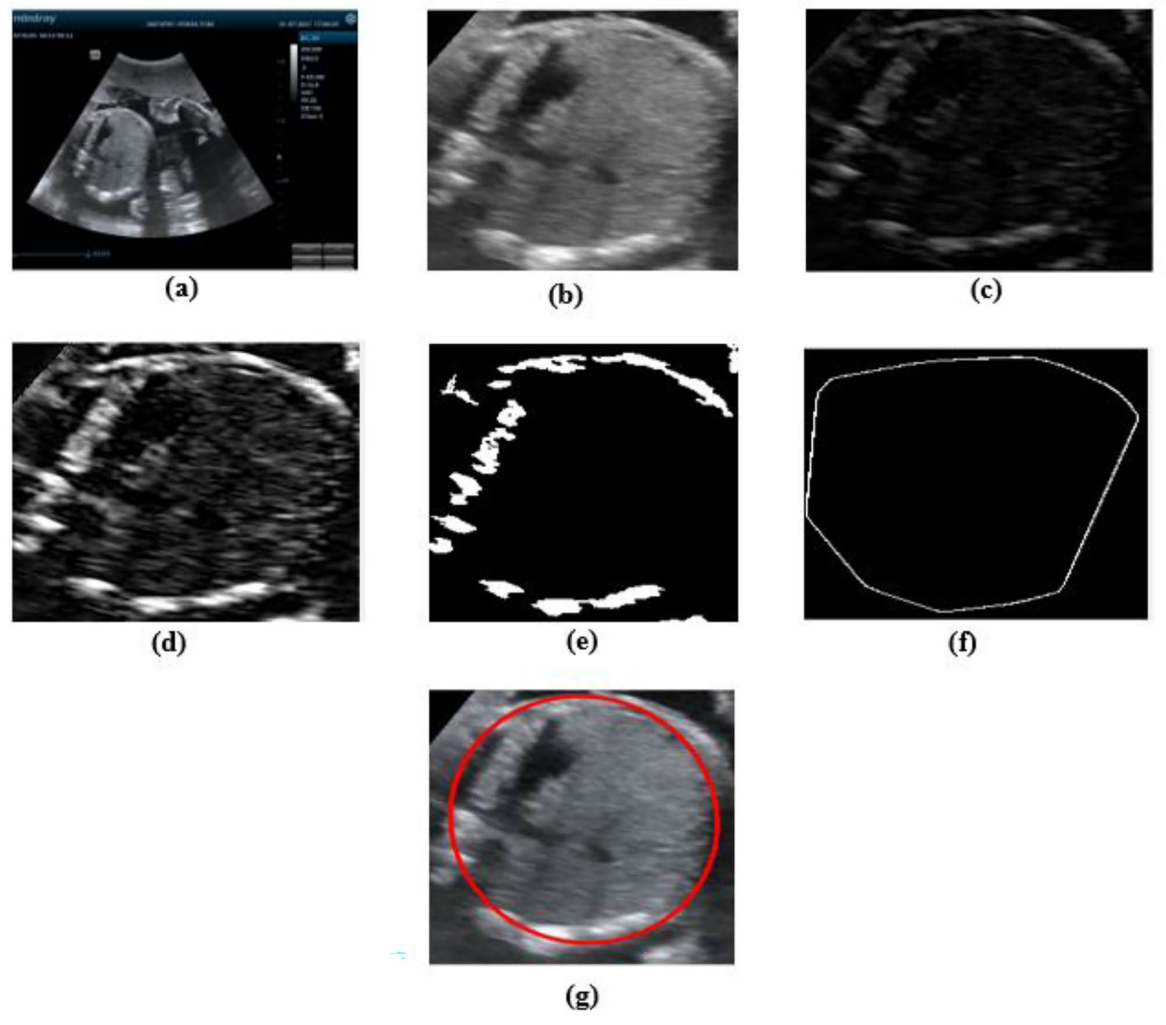
AC measurement steps, **(a)** original image, **(b)** denoised and cropped image, **(c)** morphological opened image, **(d)** contrast adjusted, **(e)** segmented image, **(f)** convex hull then edge detected image, **(g)** ellipse fitted image

In AC only the edge of the abdomen was needed so top-hat (morphological opening) and contrast adjustment were used to find the edge of the abdomen. Next segmented by adaptive thresholding technic to segment the image into background and foreground (edge of the abdomen). Then convex hull morphological analysis and canny edge detector was used to connect the discontinues edge to form complete object. Finally, ellipse was fitted onto the object edge to measure AC which is the circumference of the ellipse. Figure 3 shows the result of the image processing algorithm to measure abdominal circumference of the fetal from sample ultrasound image.

###### FL measurement

**Fig. 4:**
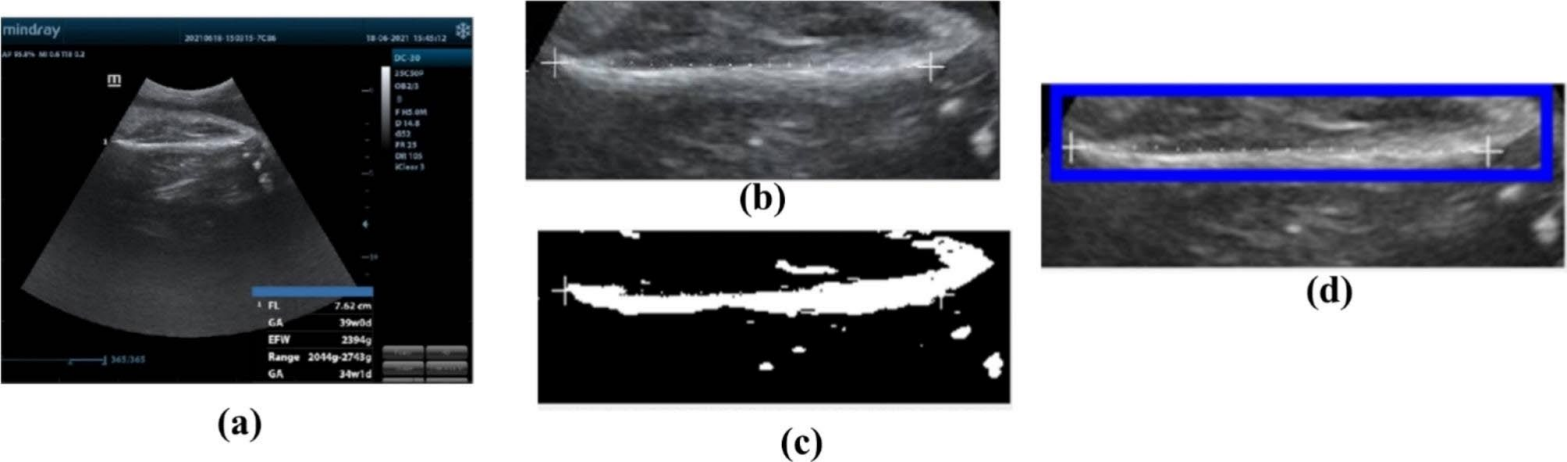
FL measurement steps, **(a)** original image, **(b)** denoised and cropped image, **(c)** segmented image, **(d)** rectangle box fitted

Acquired ultrasound image segmented by adaptive thresholding technic to segment the image into background and foreground (thigh bone). Finally, rectangle box was fitted onto the object to measure FL which is the length of the box. Figure 4 demonstrate the result of the image processing algorithm to measure femoral length of the fetal from sample ultrasound image.

#### Multiple linear regression

The multiple linear regression analysis was done to model the relationship between the dependent (actual birth weight) variable and independent variables (abdominal circumference, biparietal diameter, femoral length and gestational age). The independent variables were selected based on the value of Pearson correlation coefficient. The model statistically significantly predicted birth weight with F (4, 379) = 95.342, p (0.000) < 0.05, R^2^ = 0.502 as shown in Tables 5 and 6. Also, the model level of prediction or R is equal to 70.8% with mean estimation of 3115.62 ± 348. 167.The final regression result was FBW can be estimated by using a linear Eq. (4). The model was tested on 85 cases that undergo ultrasound assessment before less than or equal to seven days before delivery. The mean percentage error of the model was 5.89% and 78.9% estimation were within 10% percentage error (Tables 7 and 8).

4 $$\begin{gathered}EFW = - 780.532 + 7.269*AC \hfill \\\,\,\,\,\,\,\,\,\,\,\,\,\,\,\,\,\,\, - 5.031*BPD + 16.781*FL + 102.989GA \hfill \\ \end{gathered}$$

**Table 5:** Model summary (Predictors: (constant), ethnicity, abdominal circumference, gestational age. dependent variable: actual birth weight)

Model summary					
Model	R	R square	Adjusted R square	Std. error of the estimation	Durbin-Watson
1.	0.708	0.502	0.496	348.911	1.584

**Table 6:** ANOVA results (dependent variable: actual birth weight. Predictors: (constant), ethnicity, abdominal circumference, gestational age)

ANOVA						
Model		Sum of squares	df	Mean square	F	Sig.
1.	Regression	46427291.25	4	11606822.81	95.342	0.000
	Residual	46138958.75	379	121738.677		
	Total	92566250.00	383			

**Table 7:** Coefficient table (dependent variable: actual birth weight, C = Constant, AC = abdominal circumference, GA = gestational age, E = ethnicity, T = tolerance, VIF = variance of inflation factor)

Coefficient								
Model		Unstandardized coefficients		Standardized coefficients	t	Sig.	95% confidence interval for B	
		B	Std. Error	Beta			Lower bound	Upper bound
1.	C	-780.532	224.163		-3.482	0.000	-1221.29	-339.77
	AC	7.269	5.469	0.078	1.329	0.185	-3.483	18.022
	BPD	-5.031	12.939	-0.018	− 0.389	0.698	-30.473	20.410
	FL	16.781	24.460	0.033	0.686	0.493	-31.313	64.876
	GA	102.989	6.540	0.637	11.495	0.000	85.372	120.60

**Table 8:** Mean percentage error of model developed based on fetal biometry measured by image processing algorithm

Estimation	Range of percentage error	Count	Percent
Over estimation	10-15%	7	8.2%
	> 15%	3	3.5%
Estimation with accepted percentage error	< 10%	67	78.9%
Under estimation	10-15%	4	4.7%
	> 15%	4	4.7%
Total		85	100%

## Discussion

Proper and effective labor and delivery management plans for pregnant women in health facilities require the main input parameter of FBW. Factors that affect FBW include maternal ethnicity, infant sex, plurality, altitude, education, and smoking [1–7]. Typically, a normal infant birth weight ranges from 2500 to 4000 g, and deviations from this range can result in complications for both the mother and the fetus [11, 12].

During pregnancy, FBW can be estimated either by a clinical or sonographic method. The latter requires predefined formulae or models that describe birth weight as a combination of variables. However, these estimation methods can be unreliable due to the subjective nature of parameter measurement and the ineffectiveness of the models used for our country’s population.

The aim of this study was to develop a FBW estimation model tailored to the Ethiopian population, using a dataset of 484 singleton pregnant women who underwent sonographic assessments. The dataset included 1,452 fetal ultrasound images, with fetal biometry variables measured by physicians and image processing algorithms, as well as sociodemographic, obstetric, and date variables. The dataset was divided into modeling and testing subsets.

Multiple linear regression (MLR) analysis was used to develop the FBW estimation model via two approaches. The first approach incorporated fetal biometry variables measured by physicians in combination with other variables. Independent variables were selected using correlation analysis based on their strength and nature of relation with the dependent variable (actual fetal weight), and included abdominal circumference, gestational age, and maternal ethnicity.

In the second approach, an image processing algorithm proposed by the study was used to measure fetal biometry from the collected ultrasound images. Fetal biometry variables measured by this algorithm and other variables were analyzed to select variables that had a strong and positive relationship with the dependent variable (actual birth weight). The independent variables selected for MLR analysis to develop another FBW estimation model included abdominal circumference, biparietal diameter, femoral length, and gestational age.

The analysis showed that the model based on fetal biometry measured by image processing algorithm provided estimates with less than10% error in 78.9% of the estimated values during the model testing procedure, with a mean percentage error of 5.89%. In comparison, the model based on fetal biometry measured by physicians provided estimates with < 10% error in 70.61% of the estimated values, with a mean percentage error of 7.53%. The mean percentage errors were calculated from the entire test set. Additionally, as indicated in the model summary tables (Tables 3 and 7), the model based on fetal biometry measured by image processing algorithm had a higher level of prediction (R-value) than the second model. Therefore, the model based on fetal biometry measured by image processing algorithm was chosen as the final model.

This study compared newly developed models with pre-existing models for estimating FBW. A literature review identified 35 models that utilized only four commonly measured fetal biometrics as independent variables, and were developed for general fetal weight estimation (excluding models for low weight and macrocosmic fetuses). Selection criteria based on population and year of publication were used to select models for analysis. The accuracy of estimated fetal weights was compared to actual birth weights using the mean percentage error (MPE). The final new model had an MPE of 5.89%. Among the compared models, Jordaan et al. [16] and Hadlock et al. [25] had an MPE of less than 20%, while the others had an MPE between 20 and 30%. Please refer to Table 9 for more details.

**Table 9:** Comparative analysis of fetal birth estimation models (HC-head circumference, BPD- biparietal diameter, AC- abdominal circumference, FL-femoral length, GA-gestational age)

No.	Model	Fetal biometry	Population	MPE
1.	Hadlock et al. [25]	AC-BPD-HC-FL	USA	18.23%
2.	Jordaan et al. [16]	AC-BPD	South Africa	15.20%
3.	Hsieh et al. [23]	AC-BPD	China	23.59%
4.	Ferrero et al. [26]	AC-FL	Italy	22.02%
5.	Combs et al. [20]	AC-HC-FL	USA	27.44%
6.	Shinozuka et al. [14]	AC-BPD-FL	Japan	21.07%
7.	Woo et al. [24]	AC-BPD-FL	Hong Kong	22.48%
8	Waseem et al. [40]	AC-FL	Pakistan	21.81%
9	Campbell et al. [18]	AC	UK	25.69%
10.	Merz et al. [22]	AC-BPD	Germany	21.23%
11.	The current model	AC-BPD-FL-GA	Ethiopia	5.89%

In summary, our experimental results indicated that the model based on fetal biometry measured by the image processing algorithm outperformed the model based on fetal biometry measured by physicians in terms of mean percentage error and R and R2 values in the model summary of each model. Additionally, this model yielded better results compared to existing FBW estimation models.

The proposed approach holds the promise of reducing both infant and maternal mortality rates by providing precise fetal birth weight estimates, which is a pivotal factor that underpins effective, accurate, and appropriate obstetric planning, management, and decision-making. Additionally, the model can be integrated into portable devices such as point-of-care ultrasound machines, making it accessible and applicable in rural areas.

We acknowledge that the proposed model was built only using datasets gathered from South west of Ethiopia and the capital Addis Ababa. Although the model demonstrated good performance, its effectiveness could be enhanced by increasing the variability of the dataset through the collection of additional data from all regions of the country. Additionally, the study was restricted to pregnant women within the 30–42 gestational age range. Incorporating more study variables such as maternal body mass index before, during, and after pregnancy could enhance the model effectiveness. Utilizing machine learning techniques to develop models tailored to specific groups such as small and large for gestational age fetuses may also improve the model’s performance in estimating FBW for these groups.

## Conclusion

This paper presents a multiple linear regression-based model for estimating FBW of the Ethiopian population. The model was developed based on four variables: abdominal circumference, biparietal diameter, femoral length, and gestational age. With the exception of gestational age, all of these variables were measured through a custom-made automated image processing algorithm. The model was able estimate 78.9% of the fetal weights with only a mean percentage error of 5.89%. This model has practical applications in clinical settings for estimating FBW among the Ethiopian population.

## Data Availability

The datasets used and/or analysed during the current study are available from the corresponding author on reasonable request.
